# Pharmacological and Toxicological Advances in PPAR-Related Medicines

**DOI:** 10.1155/2012/940964

**Published:** 2012-10-18

**Authors:** Yuji Kamijo, Christopher J. Nicol, Stefan E. H. Alexson

**Affiliations:** ^1^Department of Nephrology, Shinshu University School of Medicine, 3-1-1 Asahi, Matsumoto 390-8621, Japan; ^2^Department of Pathology and Molecular Medicine, Cancer Biology and Genetics Division, Cancer Research Institute, and Department of Biomedical and Molecular Sciences (Pharmacology and Toxicology), Queen's University, Kingston, ON, Canada K7L 3N6; ^3^Division of Clinical Chemistry, Department of Laboratory Medicine, Karolinska Institutet, Karolinska University Hospital, 141 86 Stockholm, Sweden

Peroxisome proliferator-activated receptors (PPARs) are involved in the pathophysiology of the various types of diseases. Many types of PPAR-related medicines developed and utilized clinically all over the world exert multiple effects, including regulation of hypolipidemic, antidiabetic, anti-inflammatory, antifibrotic, and antiproliferative pathways, with emerging potential benefits in other diseases. On the other hand, these medicines may also exert various toxicities, and some PPAR drugs are no longer in use clinically because of serious complications arising in some patients. Thus, the authors here have focused on the benefits and risks of these medicines, and aim to clarify their therapeutic potential for appropriate clinical utilization. This special issue in PPAR research includes 6 review articles and 6 research articles, as follows.

**Review Articles**

The paper “*The key to unlocking the chemotherapeutic potential of PPAR*γ* ligands: Having the right combination*” by G. Skelhorne-Gross and C. J. B. Nicol is a review of the vast *in vitro, in vivo*, and human clinical trial studies, using chemotherapeutic combinations that include PPAR*γ* activating drugs. This review article reveals the novel chemotherapeutic potential of PPAR**γ** activating drugs, and provides a guide for further basic and clinical research. This information is certainly useful for optimization of chemotherapeutic interventions that will reduce the number of cancer related deaths.

The paper “*PPAR medicines and human disease: The ABCs of it all*” by A. J. Apostoli and C. J. B. Nicol is a review article that summarizes the advances of knowledge concerning effects of PPAR medicines on ATP-dependent binding cassette (ABC) transporters based on *in vitro, in vivo*, and human clinical trial studies. This review suggests the potential of PPAR-related medicines for controlling ABC transporter activity at the transcriptional level, and discusses their potential implications in human diseases with respect to cancer and atherosclerosis.

The paper “*The current knowledge of the role of PPAR in hepatic ischemia-reperfusion injury*” by M. Elias-Miró et al. is a review article concerning the roles of PPARs signaling pathways in hepatic ischemia reperfusion injury that is inherent to human liver transplantation and resection surgery. A shortage of available healthy livers for organ transplantation calls for the potential use of any available organ, including, for example, steatotic livers; however, steatotic livers are more susceptible to ischemia-reperfusion injury. This paper reviews PPAR-signaling pathways, summarizes some of the lesser known functions of PPARs in liver regeneration, and discusses potential therapies based on PPAR regulation that may minimize the observed side effects in liver surgery. This review emphasizes the need for further research into the roles of PPARs in various liver conditions and surgical procedures before being translated into treatment of human disease.

The paper “*Effects of PPAR*γ* ligands in leukemia*” by Y. Tabe et al. is a review article that describes the antitumor advances of PPAR**γ** ligands, alone and in combination with retinoic acid receptor ligands in control of cell proliferation, differentiation, and apoptosis, and discusses their potential therapeutic applications in hematological malignancies. Acute promyelocytic leukemia (APL, representing about 10% of AML patients) is unique among myeloid leukemias in that it is sensitive to all-trans-retinoic acid (ATRA). However, a number of APL patients relapse and develop ATRA resistance. This review article provides evidence on the consequences of the treatment with PPAR*γ* ligands, in particular the triterpenoid 2-cyano-3,12-dioxooleana-1,9-dien-28-oic acid (CDDO), on the epigenetic/transcriptional events induced by retinoic acid in APL cells, and supports the clinical utility of ATRA/PPAR**γ**-ligand combinations for treating hematological malignancies.

The paper “*Idealized PPAR*γ*-based therapies: Lessons from bench and bedside*” by A. A. Amato and F. de A. R. Neves is a review about the knowledge acquired regarding efficacy and safety issues by PPAR**γ** ligands. This body of work is attractive since the interest for PPAR**γ** modulation as a strategy to treat metabolic diseases has increased recently, due to better understanding of PPAR**γ** action.

The paper “*Nutraceuticals as ligands of PPAR*γ**” by M. Penumetcha and N. Santanam reviews the transcription factor PPAR*γ*, which is the target for the thiazolidinediones, the first class of PPAR*γ* agonist drugs used in the treatment of diabetes. Due to the increased adverse effects related to these drugs, newer safer drugs are being generated. This review paper describes some of the dietary components that have affinity for, and activate, PPAR*γ*, as well as their pharmacology and potential toxicology.

**Research Articles**

The paper “*PPAR*α* activation protects against anti-Thy1 nephritis by suppressing glomerular NF-*κ*B signaling*” by K. Hashimoto et al. is the first to demonstrate the glomerular protective effects of treatment using a representative PPAR*α* agonist, clofibrate, in rat mesangial proliferative glomerulonephritis model (MsPGN) anti-Thy1 nephritis. PPAR*α* activation is known to exert anti-inflammatory effects in various cells and organs through suppression of NF*κ*B signaling; however, its effect against glomerulonephritis has remained obscure. Because MsPGN is one of the significant factors leading to chronic kidney disease (CKD), the beneficial antinephritic effect of PPAR*α* activation may provide a novel treatment strategy against CKD. Their findings may also be useful to create PPAR-based therapies to treat glomerular disease.

The paper “*Hepatic cerebroside sulfotransferase is induced by PPAR*α* activation in mice*” by T. Kimura et al. is the first to examine sulfatide levels and the expression of enzymes related to sulfatide metabolism using wild-type (+/+), *Ppara*-heterozygous (+/−), and *Ppara*-null (−/−) mice given a control diet or one containing 0.1% fenofibrate, a typical PPAR*α* activator. Recent studies have revealed a protective role of serum sulfatides against arteriosclerosis and hypercoagulation. Their results suggest that PPAR*α* activation enhances hepatic sulfatide synthesis mainly through cerebroside sulfotransferase (CST) induction. Accordingly, CST may be a novel PPAR**α** target gene product candidate with implications in disease prevention and treatment.

The paper “*Fatty acid accumulation and resulting PPAR*α* activation in fibroblasts due to trifunctional protein deficiency*” by M. Wakabayashi et al. demonstrates free fatty acid accumulation, enhanced three acyl-CoA dehydrogenases, and PPAR*α* activation in the fibroblasts from six patients with mitochondrial trifunctional protein deficiency, who had abnormalities in the second through fourth reactions in fatty acid *β*-oxidation system. These novel findings suggest that the fatty acid accumulation and resulting PPAR*α* activation are major causes of the increase in the *β*-oxidation ability in the patients' fibroblasts, and that enhanced cell proliferation and increased oxidative stress relate to the development of specific clinical features. Additionally, significant suppression of the PPAR*α* activation by means of MK886 treatment may provide a new method of treating this deficiency.

In the paper “*Global gene expression profiling in PPAR*γ* agonist-treated kidneys in an orthologous rat model of human autosomal recessive polycystic kidney disease*” by D. Yoshihara et al., the authors explored the changes in gene expression by Pioglitazone (PIO), a PPAR*γ* agonist, using polycystic kidney disease (PCK) rats. By analyzing globally, they successfully found that stearoyl-coenzyme A desaturase 1 (*Scd1*) was highly expressed in PCK kidneys, and PIO decreased its expression. Notably, they found that *Scd1* plays a role in the early cystogenesis, and this is the point where PIO may intervene in the process of cystogenesis.

The paper “*Plasticizers may activate human hepatic peroxisome proliferator-activated receptor *α* less than that of a mouse but may activate constitutive androstane receptor (CAR) in liver*” by Y. Ito et al. reported the species differences concerning activation of PPAR*α* and CAR, which was induced by the oral exposure with industrial PPAR*α* ligands, including dibutyl phthalate, di(2-ethylhexyl)phthalate, and di(2-ethylhexyl)adipate, between wild-type mice and humanized PPAR*α* mice. These transcriptional species differences might cause different hepatic toxicities between murine model and human cases. This information would be valuable for the risk assessment of PPAR*α*-related medicines.

The paper “*Peroxisome proliferator-activated receptor *α* agonists differentially regulate inhibitor of DNA binding expression in rodents and human cells*” by M. del C. González et al. reported rodent versus human species differences in the regulatory manner of inhibitor of DNA binding (Id2) via PPAR*α* agonists. Since Id2 protein is involved in cell differentiation and proliferation, this finding may help to understand the species differences in toxicity of PPAR*α* agonists.

*Yuji Kamijo* *Yuji Kamijo* *Christopher J. Nicol* *Christopher J. Nicol* *Stefan E. H. Alexson* *Stefan E. H. Alexson*

